# Growth and development of soybean under changing light environments in relay intercropping system

**DOI:** 10.7717/peerj.7262

**Published:** 2019-07-23

**Authors:** Muhammad Ali Raza, Ling Yang Feng, Nasir Iqbal, Mukhtar Ahmed, Yuan Kai Chen, Muhammad Hayder Bin Khalid, Atta Mohi Ud Din, Ahsin Khan, Waqas Ijaz, Anwaar Hussain, Muhammad Atif Jamil, Muhammd Naeem, Sadam Hussain Bhutto, Muhammad Ansar, Feng Yang, Wenyu Yang

**Affiliations:** 1 College of Agronomy, Sichuan Agricultural University, Chengdu, China, China; 2 Department of Agronomy, University of Arid Agriculture Rawalpindi, Rwalpindi, Punjab, Pakistan; 3 Department of Northern Agricultural Sciences, Swedish University of Agricultural Sciences, Umea, Sweden; 4 College of Life Sciences, Sichuan Agricultural University, Yaan, China, China; 5 Chinese Academy of Agricultural Sciences, Institute of Environment and Sustainable Development in Agriculture, Beijing, China; 6 Northeast Forestry University, School of Forestry, Harbin, China

**Keywords:** Light intensity, Relay intercropping, Soybean, Light quality, Shade

## Abstract

**Background:**

Maize-soybean relay-intercropping (MS_R_) is a famous system of crop production in developing countries. However, maize shading under this system directly affects the light quality and intensity of soybean canopy. This is a challenging scenario in which to implement the MS_R_ system, in terms of varieties selection, planting pattern, and crop management since the duration of crop resource utilization clearly differs.

**Methods:**

Therefore, this experiment aimed to elucidate the effect of leaf excising treatments from maize top to fully clarify the needs and balance of light quality and intensity of intercrop-soybean under MS_R_ in field conditions. The effects of different leaf excising treatments (T0, no removal of leaves; T2, removal of two topmost leaves; T4, removal of four topmost leaves; T6, removal of six topmost leaves from maize plants were applied at first-trifoliate stage (V_1_) of soybean) on photosynthetically active radiation transmittance (PAR_T_), red to far-red ratio (R:FR), morphological and photosynthetic characteristics and total biomass production at second-trifoliate stage (V_2_), fifth-trifoliate stage (V_5_), and flowering-stage (R_1_) of soybean were investigated through field experiments for 2-years under MS_R_.

**Results:**

As compared to T0, treatment T6 increased the PAR_T_ and R:FR ratio at soybean canopy by 77% and 37% (V_2_), 70% and 34% (V_5_), and 41% and 36% (R_1_), respectively. This improved light environment in T6 considerably enhanced the leaf area index, SPAD values and photosynthetic rate of soybean plants by 66%, 25% and 49% at R_1_, respectively than T0. Similarly, relative to control, T6 also increased the stem diameter (by 29%) but decreased the plant height (by 23%) which in turn significantly increased stem breaking strength (by 87%) by reducing the lodging rate (by 59%) of soybean plants. Overall, under T6, relay-cropped soybean produced 78% of sole soybean seed-yield, and relay-cropped maize produced 81% of sole maize seed-yield. Our findings implied that by maintaining the optimum level of PAR_T_ (from 60% to 80%) and R:FR ratio (0.9 to 1.1), we can improve morphological and photosynthetic characteristics of soybean plants in MS_R_. Therefore, more attention should be paid to the light environment when considering the sustainability of MS_R_ via appropriate planting pattern selection.

## Introduction

Millions of human being in populated (China and India) and developing countries (Pakistan) live on a small scaled agricultural farm (FAO, 2013). Most of these self-supporting family farms are fronting big challenge to produce enough amount of food to fulfill the needs of increasing human-population under agricultural resources (Verschelde et al., 2013) and in changing climatic conditions (Payero et al., 2006). In the present scenario, intensification of agriculture can be used to increase crop (Phalan et al., 2011). To ensure high crop, it is important to follow optimum agriculture practices like the selection of suitable cropping systems and area-specific varieties which have the ability to utilize sun-light and land resources more efficiently (Raza et al., 2018a, 2018b; Zhang et al., 2015). Intercropping is the system of growing two or more crops in the same area (Lithourgidis et al., 2011). As compared to mono-cropping, intercropping considerably increases the crop yield by effective utilization of land and water (Inal et al., 2007). Relay-intercropping of legumes with cereals is the well-known practice in many countries (Dhima et al., 2007), is suggested to be used for its overall economic profitability, suppression of insect, pests, and weeds, environment-friendly services and high productivity (Echarte et al., 2011). Maize-soybean relay-intercropping system (MS_R_), is one of the main types of intercropping systems and widely practiced in areas where crop season is very short for two crops (Coll et al., 2012; Monzon et al., 2014; Yang et al., 2015).

In MS_R_, maize is normally sown in narrow-double rows in April and harvested in August. The soybean is seeded in wide-double rows in June and harvested in October (Yang et al., 2014). Under this system, the reproductive phase of maize and seedling phase of soybean crop overlap approximately for 9 to 10 weeks. Thus, this system can be used for agricultural production where the planting season for double crops is too small. Furthermore, MS_R_ enhances the soil productivity due to the fixation of nitrogen by soybean which in turn decrease the requirement of nitrogen fertilizers (Stern, 1993). However, soybean plants are extremely responsive to shading conditions (Wolff & Coltman, 1990) and soybean plants in MS_R_ suffered from maize shading during their vegetative growth period that increased the plant height and it became more vulnerable to lodging as shade increases. Lodging in plants inhibits the transportation of photo-assimilates, nutrients, and water which in turn reduces the crop yield (Li et al., 2014; Zuber & Kang, 1978). Soybean lodging is a major problem of MS_R_ (Liu et al., 2015), therefore, to investigate further how we can reduce the lodging rate of soybean plants during the co-growth period in MS_R_ is an important research question.

Light is the most important abiotic factors for plant growth (Yang et al., 2018b), and any change in the light environment (light quality and quantity) brings significant changes in the morphology and physiology of soybean plants (Wu, Gong & Yang, 2017). Shading conditions negatively affect the central processes of plants such as leaf growth, photosynthesis, and biomass production (Kong et al., 2016; Wu et al., 2018). Similarly, shading disturbs the carbon status of crops because the demand for photo-assimilate accelerated while its production reduces (Lichtenthaler et al., 1981; Su et al., 2014). Additionally, the pattern of photo-assimilates utilization into expensive operations, such as the production of protective proteins enhances with under heavy shading environments (Yang et al., 2018a). However, previously it has been reported that plant tolerance to shading is improved at a higher photosynthetic rate, adequate and uninterrupted light availability should be considered to investigate the response of plants to shading (Rijkers, Pons & Bongers, 2000). Crop biomass accumulation is mainly dependent on the current rate of photosynthesis (Feng et al., 2018), and shading conditions significantly lowers the photosynthetic rate of soybean plants (Yang et al., 2018a) which decreased the leaf area and biomass accumulation of soybean plants in MS_R_ (Ahmed et al., 2018; Khalid et al., 2019). Overall, these results suggest a close relationship between photosynthetic rate and available light (Feng et al., 2018). Therefore, it is vital to study the effect of changing light environment on soybean photosynthesis in field conditions to understand the photosynthetic process of soybean plants in the MS_R_ system.

Thus, in this present study leaves were removed from maize canopy to study the impacts of increasing photosynthetically active radiation transmittance (PAR_T_) on soybean growth in MS_R_. The aims of the present study were: (a) to determine the impact of leaf excising on light quantity and quality at soybean canopy in MS_R_; (b) to investigate the effect of this change in light environment on the morphology, physiology, and biomass production of soybean under MS_R_. This experiment provides new insight to improve the seedling growth of soybean plants in MS_R_. The outcomes will be useful for developing innovative agronomic practices or planting patterns for the betterment of soybean growth during the co-growth period in MS_R_.

## Materials and Methods

### Research location and planting material

Field trails were carried out from April to November in 2017 and 2018 at the Modern Research Farm of Sichuan Agricultural University, Yaan (29°59′N, 103°00′E, altitude 620 m), Sichuan Province, P. R. China. The semi-compact cultivar of maize (*Zea mays* L.) Zhenghong-505 and shade-tolerant cultivar of soybean (*Glycine max* L.) Nandou-12 was used in both years. These are the major summer cultivars of maize and soybean and are extensively used in Southwest of China (Liu et al., 2016).

### Weather and soil characteristics

The experimental site has a humid climatic condition with an average annual temperature of 16 °C and rainfall 1,200 mm. The weather data which includes monthly rainfall, average temperature, humidity, and wind speed during the planting seasons from 2017 to 2018 is presented in Table 1. Every year, disk plough was used to plough the field. The physiochemical characteristics of soil at Yaan are: pH = 6.6, organic matter 29.6-gram kilogram^−1^, total N = 29.8-gram kilogram^−1^, = 1.6-gram kilogram^−1^, total P = 1.28-gram kilogram^−1^, total K = 16.3-gram kilogram^−1^, available N = 317.1 milligram kilogram^−1^, available P = 42.2 milligram kilogram^−1^, and available K = 382.1 milligram kilogram^−1^, in 0–20 cm soil layer.

**Table 1:** Monthly rainfall, average temperature, humidity, and wind speed from March to October in the growing seasons of 2017 and 2018.

Month	Years							
	2017				2018			
	Rainfall (mm)	Average T (°C)	Humidity (%)	Wind speed (m s^−1^)	Rainfall (mm)	Average T (°C)	Humidity (%)	Wind speed (m s^−1^)
March	41.1	15.63	56.34	0.31	26.7	14.11	55.37	0.31
April	65.5	19.39	62.27	0.47	53.5	19.14	57.31	0.44
May	93.7	22.45	66.31	0.55	113.1	23.52	56.36	0.56
June	167.1	26.41	61.37	0.43	151.7	25.54	56.45	0.43
July	205.7	27.73	84.43	0.75	185.4	29.19	62.39	0.39
August	126.8	28.66	65.99	0.61	223.6	27.72	80.14	1.26
September	172.5	22.32	79.21	0.87	146.3	23.55	54.35	0.82
October	21.4	19.48	57.29	0.42	59.4	17.68	77.87	0.49
March–October	893.8	22.75	66.65	0.55	959.7	22.56	62.53	0.59

### Experimental design and details

**Figure 1:**
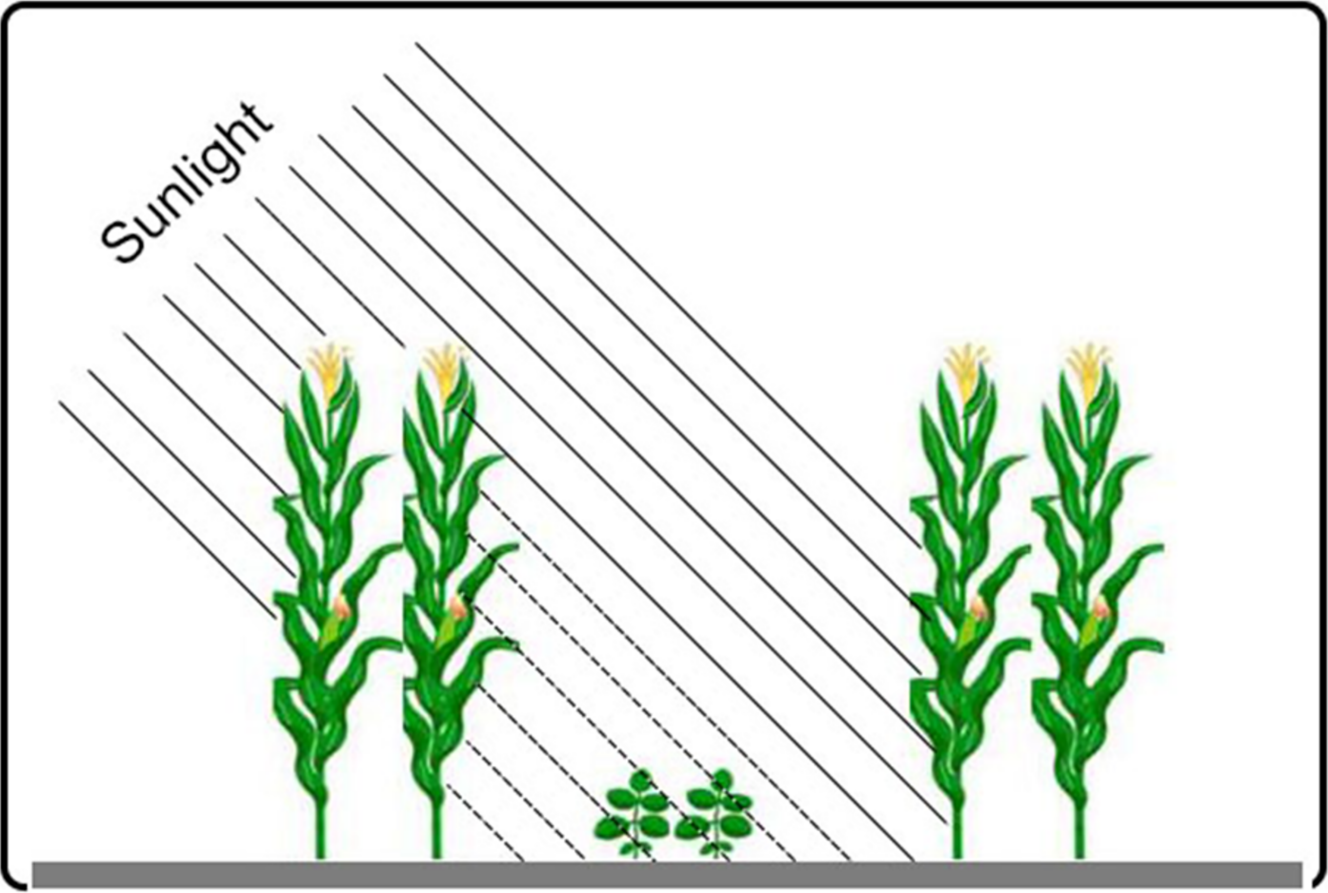
Schematic representation of the maize-soybean relay-intercropping system considered in the present experiment.

In this field experiment, a randomized block design was used, with six treatments and three replicates. The MS_R_ was used in this study. Within the MS_R_—described here as the introduction of soybean rows between the rows of maize at tasseling stage—presence of maize plants adds complexity in terms of spatiotemporal (light intensity and quality) dynamics for resource-use (Fig. 1). The MS_R_ used narrow-wide row planting pattern with alternating strips of maize and soybean. Every strip in MS_R_ contains two maize rows and two soybean rows (2:2). Row to row distance between maize to maize and soybean to soybean row was 40 cm, and 60 cm distance was maintained between maize and soybean. For sole soybean (SS), 50 cm distance was kept between the rows, and for sole maize SM, 70 cm distance was kept between the maize rows. The size of each experimental plot was 6 × 6 m. Both varieties were overseeded and thinned to keep the planting density of six plants m^−2^ for maize and 10 plants m^−2^ for soybean in MS_R_, and similar planting density of maize (six plants m^−2^) and soybean (six plants m^−2^) was kept in SS and SM. The maize crop was sown in the second week of April in 2017 and 2018, and harvested in the first week of August 2017 and 2018. Soybean was sown on in the second week of June 2017 and 2018 and harvested in the last week of October 2017 and 2018. Basal nitrogen at 135 kg ha^−1^ as urea, phosphorus at 72 kg ha^−1^ as calcium superphosphate, and potassium at 90 kg ha^−1^ as potassium sulfate were applied in MS_R_ and SM. At the V_6_ stage of maize, the second dose of nitrogen for maize plants was applied at 75 kg ha^−1^ in all maize rows. The nitrogen, phosphorus, and potassium at 75, 40, and four kg ha^−1^ as urea, calcium superphosphate, and potassium sulfate, respectively were basally applied for soybean, and both crops were grown on rainfall water.

### Treatments

**Figure 2:**
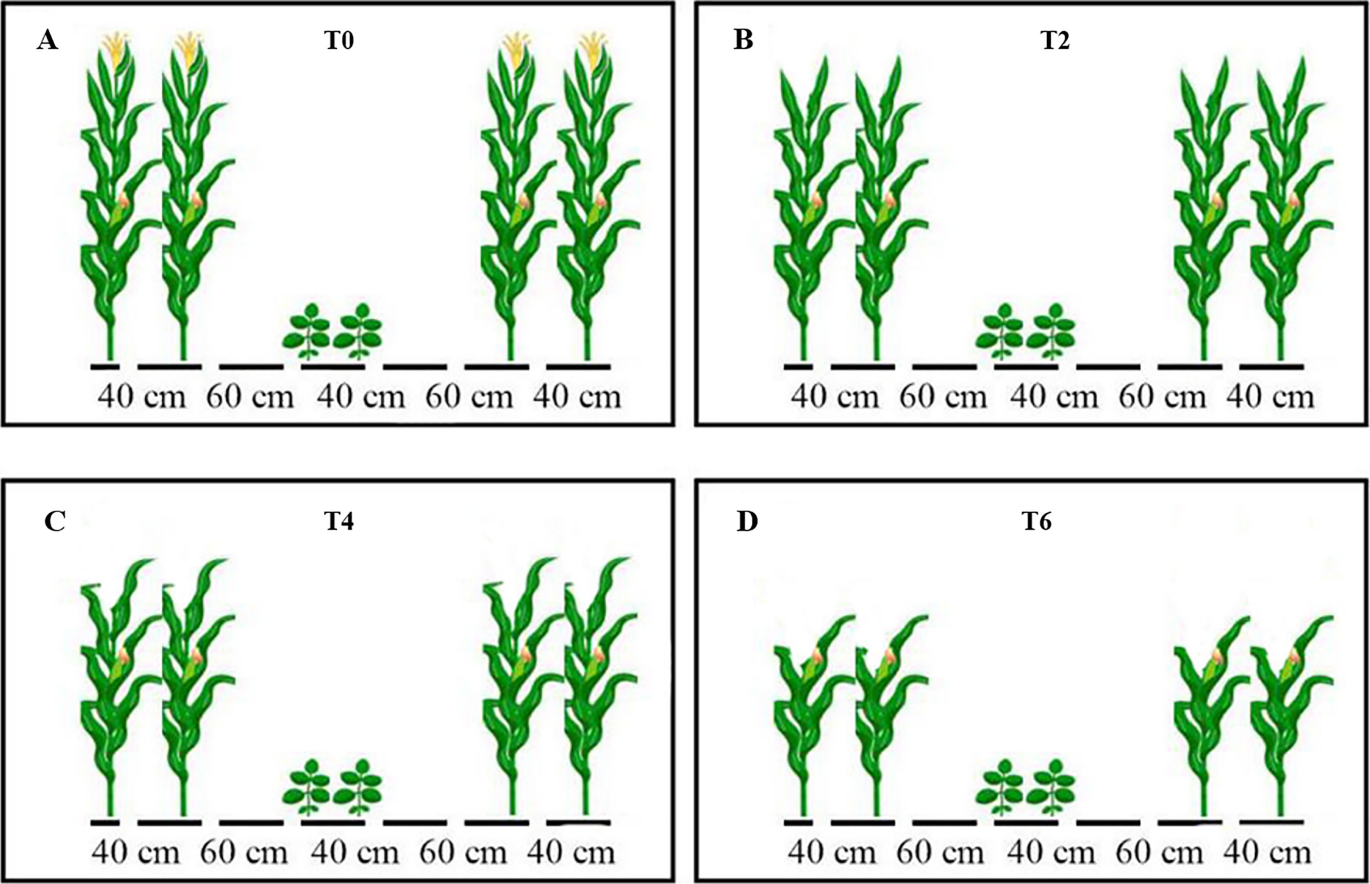
Schematic representation of maize canopy as affected by leaf excising treatments from 2017 to 2018 growing season. The T0 (A), T2 (B), T4 (C), and T6 (D) represent the no removal, removal of two, four, or six leaves, respectively, from maize canopy at V_1_ stage of soybean under relay-intercropping system.

Maize crop was grown up to the silking stage (June 30, 2017 and June 28, 2018) of maize, then following leaf removal treatments by cutting different numbers of leaves from the top of maize were set up in MS_R_ to change the light environment (quantity and quality) at soybean canopy (Fig. 2): (1) T0 (no removal of leaves); (2) T2 (removal of topmost two leaves from maize plants); (3) T4 (removal of topmost four leaves from maize plants); (4) T6 (removal of topmost six leaves from maize plants); (5) SS sole cropping of soybean and (6) SM sole cropping of maize. In addition, the soybean was at V_1_ stage when leaf excising treatments were applied.

### Measurements

#### Light environment

The measurement of photosynthetically active radiation (PAR) in all treatments (leaf removal treatments in MS_R_ and SS) was done to illustrate the changes in the light environment of soybean seedlings. The PAR in different treatments of MS_R_ and SS was determined at the second-trifoliate stage (V_2_), fifth-trifoliate stage (V_5_), and flowering stage (R_1_) of soybean in 2017 and 2018. For this purpose, the sensors of the light measuring-instrument were placed on the horizontal arm of the observing scaffold, which was 30 cm above the ground level. All the measurements were performed every 10 cm between the maize rows of different treatments using LI-191SA quantum sensors (LI-COR Inc., Lincoln, NE, USA) with LI-1400 data logger. Additionally, the incident PAR was simultaneously determined using LI-190SA quantum sensors (LI-COR Inc., Lincoln, NE, USA) at the top of maize canopy under MS_R_. The PAR of each treatment was measured thrice from 10:00 am to 12:00 am on a clear day and average was estimated. Then the PAR_T_ at soybean plants was determined by using the following formula (Serrano, Gamon & Peñuelas, 2000):

$${\rm{PAR\, Transmittance}} = {{Is} \over {Im}} \times 100$$

Where *Is* and *Im* are the PAR at the top of soybean and maize top.

After the determination of light intensity, changes in light quality (red to far-red ratio (R:FR)) was measured at V_2_, V_5_, and R_1_ of soybean by using HR350 (Hipoint Inc., Gaoxiong, Taiwan) from 360 to 760 nm. The red to far-red light ratio was determined by dividing the red light (R, 655–665 nm) by the far-red light (FR, 725–735 nm) (Hertel et al., 2012).

#### SPAD values and photosynthetic characteristics

SPAD values of soybean leaves were measured by using SPAD 502 Minolta chlorophyll meter at V_2_, V_5_, and R_1_ from all experimental plots in both years. Li-6400 portable photosynthesis-system (LI-COR Inc., Lincoln, NE, USA) equipped with LED leaf-chamber was utilized for photosynthetic characteristics measurement of soybean leaves. The photosynthetic characteristics (stomatal conductance (Gs), intercellular CO_2_ concentration (Ci), transpiration-rate (Tr) and photosynthetic-rate (Pn)) were determined under flow rate of 500 μmol s^−1^, steady light-intensity of 800 (µmol m^−2^ s^−1^), vapor pressure deficit of 3.5 mmol mol^−1^, environment temperature of 25 °C and a CO_2_ concentration of 400 (µmol mol^−1^) (Yang et al., 2017). In addition, three expanded leaves of soybean plants were chosen at V_2_, V_5_, and R_1_, and photosynthetic characteristics were measured from all treatments. Measurements of photosynthetic characteristics were done from 10:00 am to 11:00 am on a clear day.

#### Morphological parameters

At V_2_, V_5_, and R_1_, 20 soybean seedlings from the middle rows of every treatment were selected, the plant height was measured from base to top and Vernier caliper was used to determine the stem diameter. Lodging rate of soybean plants was measured by the following procedure (Liu et al., 2016): when the angle between soybean stem and ground was less than 30° it was considered as a lodged plant. The basal internode was used to determine the stem breaking strength of soybean plants by using the digital plant lodging tester according to the previously described method (Liu et al., 2015).

#### Leaf area index and total biomass accumulation

The leaf area index (LAI), total biomass accumulation and distribution in different plant parts were measured at V_2_, V_5_, and R_1_. A total of 10 soybean plants from each treatment were sampled destructively, and the maximum leaf width and length were measured by using a ruler. Leaf area was determined by multiplying the leaf-width, leaf-length, and crop-specific co-efficient factor of 0.75 for soybean (Gao et al., 2010). For total accumulation of biomass and its distribution in various plant parts, 20 consecutive soybean plants were destructively sampled from every treatment at V_2_, V_5_, and R_1_. After that all the sampled plants were divided into various plant parts of soybean plants (root, stem, and leaves) and placed it in oven for 1 hat 105 °C to kill the fresh-tissues and dried at 65 °C to get the constant weight before final weighing of each plant part of soybean for total biomass accumulation and distribution analysis.

#### Maize yield

To assess the impact of leaf excising treatments on seed yield of maize and soybean under MS_R_. At maturity, four m^2^ plants of both crops (maize and soybean) were collected from each treatment. Then sampled plants of maize and soybean were sun-dried for 6 days. After that, the dried maize and soybean plants were threshed and weighed to measure the seed yields (kg ha^−1^) of maize and soybean plants under all treatments.

#### Statistical analysis

All the obtained data for each parameter was analyzed by using Statistix 8.1 (Raza et al., 2018b). The ANOVA technique and least significant difference (LSD) test were used to measure the impact of leaf excising treatments on the light environment, morphological characteristics, SPAD values, photosynthetic parameters, LAI, total biomass accumulation and its distribution, and maize seed yield. All the means were compared at 5% probability level (Steel & Torrie, 1960).

## Results

### Light quality and intensity

At R_1_, the mean values for PAR and PAR_T_ showed that different leaf removal treatments had a significant impact on PAR and PAR_T_ at soybean canopy, while year showed non-significant effect on PAR and PAR_T_ (*P* < 0.05). Similarly, there was non-significant interaction of year × leaf excising treatments at all sampling stages (Table 2). In both years, plants in SS always obtained the higher PAR and PAR_T_ than those under T0, T2, T4, and T6 in MS_R_. However, the leaf removal treatments increased the PAR at soybean top in MS_R_. Compared to T0, the PAR in T2, T4, and T6 at soybean canopy increased by 24%, 51%, and 77% (V_2_), 27%, 46%, and 71% (V_5_), and 17%, 31%, and 41% (R_1_) respectively, their PAR_T_ were 36%, 55%, and 65% (V_2_), 42%, 53%, and 71% (V_5_), and 61%, 72%, and 86% (R_1_) of SS, respectively (mean values in 2017 and 2018). Furthermore, PAR and PAR_T_ showed a similar trend in the following order: T6 > T4 > T2 > T0.

**Table 2:** Effects of leaf excising treatments on incident-PAR and PAR-transmittance of soybean canopy at second-trifoliate stage (V_2_), fifth-trifoliate stage (V_5_), and flowering-stage (R_1_) under sole cropping and relay intercropping system from 2017 to 2018.

Years	Treatments	Growth stages					
		V_2_		V_5_		R_1_	
		PAR (µmol m^−2^ s^−1^)	PAR_T_ (%)	PAR (µmol m^−2^ s^−1^)	PAR_T_ (%)	PAR (µmol m^−2^ s^−1^)	PAR_T_ (%)
2017	T0	556.7^e^	33.0^e^	675.2^d^	39.6^d^	997.9^d^	59.2^d^
	T2	728.5^d^	43.2^d^	849.9^c^	49.9^c^	1,162.5^c^	69.1^c^
	T4	859.2^c^	50.9^c^	983.4^c^	57.8^c^	1,308.8^b^	77.8^b^
	T6	1,052.9^b^	62.4^b^	1,180.7^b^	69.2^b^	1,402.9^b^	83.4^b^
	SS	1,687.7^a^	100^a^	1,703.6^a^	100^a^	1,680.7^a^	100^a^
	LSD	102.75	5.72	150.80	8.39	115.93	6.98
2018	T0	636.9^c^	38.9^c^	695.8^d^	41.9^d^	1,026.2^e^	62.2^e^
	T2	751.0^c^	45.9^c^	887.2^b^	53.3^c^	1,210.0^d^	73.4^d^
	T4	942.0^b^	57.6^b^	1,016.3^bc^	61.0^bc^	1,336.2^c^	81.1^c^
	T6	1,063.9^b^	65.1^b^	1,164.2^b^	69.7^b^	1,450.9^b^	88.0^b^
	SS	1,636.8^a^	100^a^	1,667.2^a^	100^a^	1,648.3^a^	100^a^
	LSD	144.60	8.83	167.59	9.41	77.24	4.57
ANOVA							
Year (Y)		NS	NS	NS	NS	*	NS
Treatments (T)		*	*	*	*	*	*
Interaction (Y × T)		NS	NS	NS	NS	NS	NS

**Notes:**

The PAR refers to photosynthetically active radiations, and T0, T2, T4, and T6 represent the no removal, removal of two, four, or six leaves, respectively, from maize canopy under relay-intercropping system. The SS refer to sole cropping system of soybean. Means are averaged over three replicates. Means that do not share the same letters in the column differ significantly at *P* ≤ 0.05.

NS, non-significant; *, significant.

**Figure 3:**
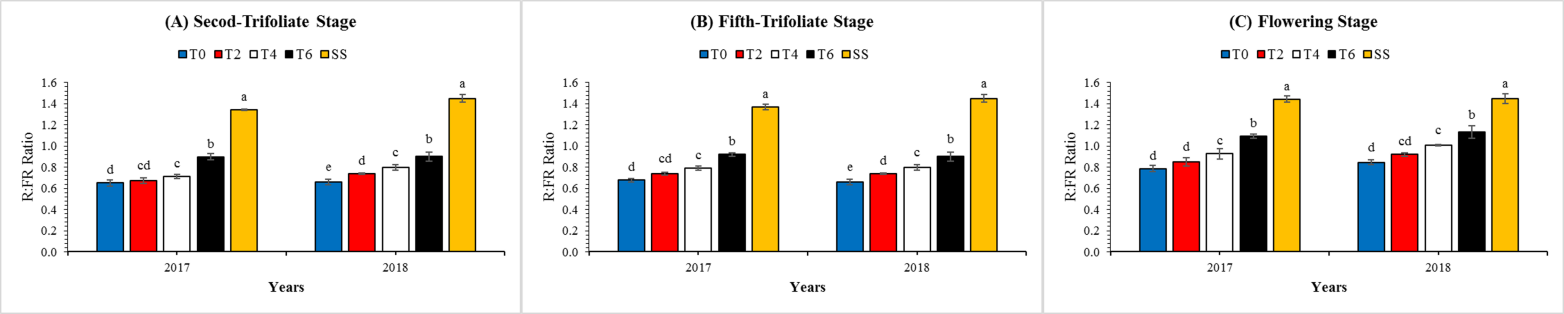
Changes in red to far red ratio (light quality) of the soybean canopy as affected by leaf excising treatments from 2017 to 2018 growing season. (A), (B) and (C) refer to second-trifoliate stage (V_2_), fifth-trifoliate stage (V_5_), and flowering-stage (R_1_), respectively. The T0, T2, T4, and T6 represent the no removal, removal of two, four, or six leaves, respectively, from maize canopy under relay-intercropping system. The SS refer to sole cropping system of soybean. Means are averaged over three replicates. Bars show ± standard errors, (*n* = 3). Within a bar, different lowercase letters show a significant difference (*P* ≤ 0.05) between treatments.

The ratios of red (R) to far-red (FR) light at soybean top under SS and MS_R_ were measured, as presented in Fig. 3. Different leaf excising treatments and years had a significant and non-significant (*P* < 0.05) effect on the R:FR ratio, respectively (Table 3). Under SS, the highest value of R:FR ratio was 1.46 at V_5_, while the lowest value was 1.40 at V_2_ in both years. For MS_R_, the maximum R:FR ratios 1.11, 1.16, and 1.35 at V_2_, V_5_ and R_1_, respectively, were measured in T6, whereas minimum values 0.84, 0.90, 1.03 at V_2_, V_5_, and R_1_, respectively, were noted under T0. However, the differences in R:FR ratios between sole-cropped and relay-cropped soybean were decreased from T0 to T6. Furthermore, the interactive effect of year and leaf excising treatments was found significant and non-significant at V_2_, and V_5_ and R_1_, respectively (Table 3).

**Table 3:** ANOVA results for effects of years (Y), leaf excising treatments (T) and their interactions (Y × T) on red to far red ratio, stem breaking strength (*N*), stem diameter (mm) and leaf area index at second-trifoliate stage (V_2_), fifth-trifoliate stage (V_5_), and flowering-stage (R_1_), and seed yield (kg ha^−1^) of maize and soybean under sole cropping and relay intercropping system from 2017 to 2018.

Factors	Red to far ratio			Stem breaking strength			Stem diameter			Leaf area index			Seed yield	
	V_2_	V_5_	R_1_	V_2_	V_5_	R_1_	V_2_	V_5_	R_1_	V_2_	V_5_	R_1_	Maize	Soybean
Years (Y)	NS	NS	NS	NS	*	NS	*	*	*	NS	NS	*	*	*
Treatments (T)	*	*	*	*	*	*	*	*	*	*	*	*	*	*
Interaction (Y × T)	*	*	NS	NS	NS	NS	NS	NS	*	*	NS	NS	NS	*

**Notes:**

Significance level (*P < 0.05).

NS, non-significant; *, significant.

### SPAD values and photosynthetic parameters

The leaf SPAD values from V_2_ stage (i.e., two fully trifoliate) to R_1_ stage (i.e., start of flowering) of soybean leaves are showed in Table 4. Leaf excising treatments significantly (*P* < 0.05) affected the SPAD vales of soybean leaves at all stages. At the V_2_ stage, the SPAD values of soybean leaves were similar in all treatments but was quickly differentiating thereafter. At R_1_ (third stage of measurement), among all the treatments, SS had achieved their maximum SPAD values. However, in MS_R_, the highest SPAD values were noticed under treatment T6 followed by T4, T2, and T0 in both years. Moreover, the interactive effect of leaf excising treatments and year for SPAD values was found non-significant at V_2_, V_5_, and R_1_ (Table 4).

**Table 4:** Effects of leaf excising treatments on SPAD values, plant height (cm), lodging rate (%) and total biomass (g plant^−1^) of soybean plants at second-trifoliate stage (V_2_), fifth-trifoliate stage (V_5_), and flowering-stage (R_1_) under sole cropping and relay intercropping system from 2017 to 2018.

Years	Treatments	SPAD values			Plant height (cm)			Lodging rate (%)			Total biomass (g plant^−1^)		
		V_2_	V_5_	R_1_	V_2_	V_5_	R_1_	V_2_	V_5_	R_1_	V_2_	V_5_	R_1_
2017	T0	24.7^NS^	26.8^d^	29.2^d^	19.8^a^	43.7^a^	57.6^a^	13.3^NS^	36.7^a^	43.3^a^	1.11^d^	4.4^d^	10.3^c^
	T2	25.1	29.6^cd^	32.0^c^	19.6^a^	42.1^a^	56.0^ab^	11.7	28.3^ab^	35.1^ab^	1.26^c^	4.8^d^	12.5^c^
	T4	25.5	31.0^bc^	34.4^c^	18.4^b^	38.8^b^	53.0^b^	10.1	23.3^b^	28.3^bc^	1.33^bc^	5.4^c^	15.7^b^
	T6	25.7	33.7^ab^	37.6^b^	17.3^c^	32.4^c^	46.4^c^	8.3	16.6^bc^	21.7^cd^	1.38^b^	6.1^b^	18.7^b^
	SS	25.8	36.5^a^	40.9^a^	15.1^d^	28.3^d^	40.1^d^	5.1	8.3^c^	13.3^d^	1.63^a^	7.3^a^	24.0^a^
	LSD	1.87	2.86	2.63	1.08	2.32	3.17	12.03	12.10	11.01	0.07	0.52	3.07
2018	T0	25.1^NS^	29.1^d^	31.1^c^	22.1^a^	51.9^a^	68.7^a^	11.7^NS^	28.3^a^	38.3^a^	1.35^e^	4.8^c^	12.5^d^
	T2	25.3	30.8^cd^	33.0^c^	20.2^ab^	47.9^b^	63.9^a^	10.1	23.3^a^	31.7^ab^	1.50^d^	5.2^c^	13.6^d^
	T4	25.4	32.6^c^	34.8^bc^	18.9^bc^	44.1^c^	56.4^b^	8.3	21.6^a^	23.3^bc^	1.67^c^	6.1^b^	17.3^c^
	T6	25.7	36.4^b^	37.9^b^	17.8^bc^	38.4^b^	51.2^c^	6.7	13.3^b^	11.7^c^	1.80^b^	6.8^b^	21.5^b^
	SS	26.4	40.5^a^	43.3^a^	16.3^c^	33.1^e^	43.5^d^	3.3	6.7^b^	10^c^	1.97^a^	7.9^a^	25.7^a^
	LSD	2.84	2.56	4.07	2.72	1.31	5.21	10.67	7.59	13.91	0.06	0.73	3.19
ANOVA													
Year (Y)		NS	NS	NS	NS	*	*	NS	NS	NS	*	*	NS
Treatments (T)		NS	*	*	*	*	*	NS	*	*	*	*	*
Interaction (Y × T)		NS	NS	NS	NS	NS	*	NS	NS	NS	*	NS	NS

**Notes:**

The T0, T2, T4, and T6 represent the no removal, removal of two, four, or six leaves, respectively, from maize canopy under relay-intercropping system. The SS and SM refer to sole cropping system of soybean and maize, respectively. Means are averaged over three replicates. Means that do not share the same letters in the column differ significantly at *P* ≤ 0.05.

NS, non-significant; *, significant.

Compared with T0, Pn, and Tr increased significantly under leaf removal treatments (T2, T4, and T6), while Gs and Ci of soybean leaves were decreased at V_2_, V_5_, and R_1_ (Table 5). The photosynthetic rate of T2, T4, and T6, respectively increased by 7%, 20%, and 47% at V_2_, 10%, 24%, and 34% at V_5_, and 11%, 23%, and 33% at R_1_ than those under T0 in MS_R_ (Table 5). Moreover, the photosynthetic characteristics of soybean leaves at R_1_ followed a similar trend to that at V_2_ and V_5_ stages, whereas Pn under T4 and T6 considerably increased. Interestingly, the Pn of soybean leaves at V_5_ and R_1_ under T6 did not differ significantly from that of under SS (Table 5). In addition, the interactive effect of leaf excising treatments and year for Pn, Gs, and Tr was found significant, while for Ci it was found significant at all stages.

**Table 5:** Effects of leaf excising treatments on photosynthetic characteristics of soybean canopy at second-trifoliate stage (V_2_), fifth-trifoliate stage (V_5_), and flowering-stage (R_1_) under sole cropping and relay intercropping system from 2017 to 2018.

Years	Treatments	Photosynthetic rate (μmol CO_2_ m^−2^ s^−1^)			Stomatal conductance (mol H_2_O m^−2^ s^−1^)			Transpiration rate (mmol H_2_O m^−2^ s^−1^)			Intercellular CO_2_ concentration (μmol CO_2_ m^−2^ s^−1^)		
		V_2_	V_5_	R_1_	V_2_	V_5_	R_1_	V_2_	V_5_	R_1_	V_2_	V_5_	R_1_
2017	T0	7.0^e^	11.4^d^	12.8^d^	0.31^a^	0.38^a^	0.62^a^	1.99^d^	2.51^d^	3.40^e^	326.5^a^	368.3^a^	349.5^a^
	T2	7.8^d^	12.5^c^	14.4^c^	0.24^b^	0.35^ab^	0.54^b^	2.04^cd^	2.60^d^	3.69^d^	321.9^a^	359.2^a^	331.0^b^
	T4	8.6^c^	14.2^b^	16.1^b^	0.22^b^	0.30^bc^	0.49^bc^	2.15^c^	2.81^c^	3.96^c^	304.7^a^	344.9^a^	320.9^b^
	T6	10.9^b^	17.0^a^	19.2^a^	0.18^a^	0.27^cd^	0.44^c^	2.39^b^	3.02^b^	4.30^b^	282.5^b^	319.7^b^	291.1^c^
	SS	13.1^a^	17.1^a^	19.9^a^	0.217^a^	0.24^d^	0.35^d^	2.61^a^	3.17^a^	4.76^a^	250.4^c^	279.1^a^	271.0^d^
	LSD	0.58	1.10	0.93	0.01	0.05	0.08	0.12	0.13	0.10	21.87	24.96	14.81
2018	T0	8.2^d^	12.3^d^	14.3^d^	0.33^a^	0.44^a^	0.73^a^	2.36^c^	2.53^d^	3.52^d^	284.6^a^	310.7^a^	430.2^a^
	T2	8.5^d^	13.6^c^	15.6^c^	0.30^ab^	0.40^ab^	0.59^ab^	2.72^c^	2.75^d^	3.65^cd^	267.9^b^	292.3^b^	397.7^b^
	T4	9.6^c^	15.2^b^	17.2^b^	0.27^bc^	0.38^b^	0.54^b^	2.93^c^	3.37^c^	3.84^c^	246.1^c^	264.1^c^	383.1^b^
	T6	11.6^b^	18.1^a^	21.1^a^	0.25^c^	0.33^c^	0.47^bc^	3.89^b^	4.06^b^	4.47^b^	239.7^c^	243.5^d^	359.7^c^
	SS	14.3^a^	18.5^a^	21.4^a^	0.24^c^	0.26^d^	0.35^c^	4.68^a^	4.79^a^	5.25^a^	212.4^d^	231.1^d^	327.3^d^
	LSD	0.56	1.07	0.18*	0.04	0.05	0.06	0.62	0.56	0.11	12.75	17.00	16.63
ANOVA													
Year (Y)		*	*	*	NS	NS	NS	NS	*	NS	*	NS	NS
Treatments (T)		*	*	*	*	*	*	*	*	*	*	*	*
Interaction (Y × T)		NS	NS	NS	NS	NS	NS	NS	NS	NS	*	*	*

**Notes:**

The T0, T2, T4, and T6 represent the no removal, removal of two, four, or six leaves, respectively, from maize canopy under relay-intercropping system. The SS refer to sole cropping system of soybean. Means are averaged over three replicates. Means that do not share the same letters in the column differ significantly at *P* ≤ 0.05.

NS, non-significant; *, significant.

### Morphological characteristics

**Figure 4:**
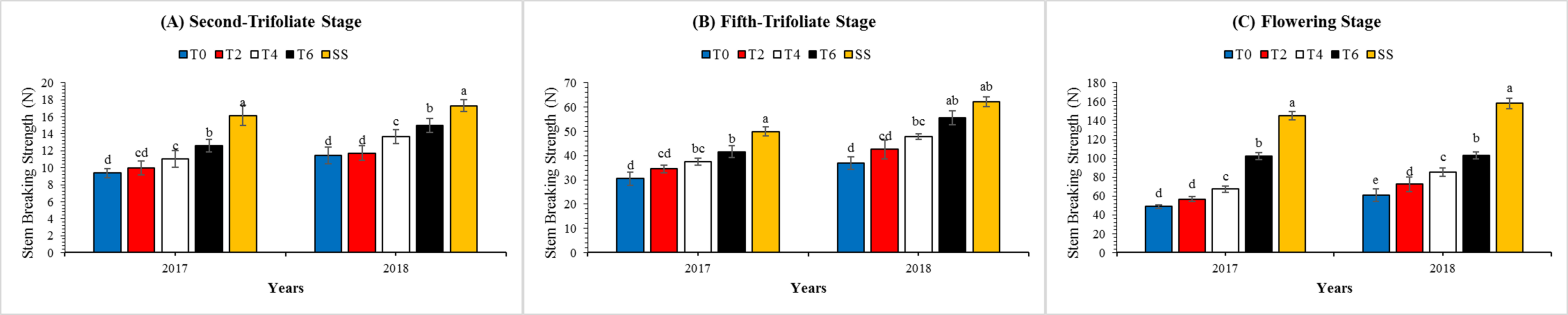
Changes in the stem breaking strength of soybean plants as affected by leaf excising treatments during 2017 to 2018 growing season. (A), (B) and (C) refer to second-trifoliate stage (V_2_), fifth-trifoliate stage (V_5_), and flowering-stage (R_1_), respectively. The T0, T2, T4, and T6 represent the no removal, removal of two, four, or six leaves, respectively, from maize canopy under relay-intercropping system. The SS refer to sole cropping system of soybean. Means are averaged over three replicates. Bars show ± standard errors, (*n* = 3). Within a bar, different lowercase letters show a significant difference (*P* ≤ 0.05) between treatments.

**Figure 5:**
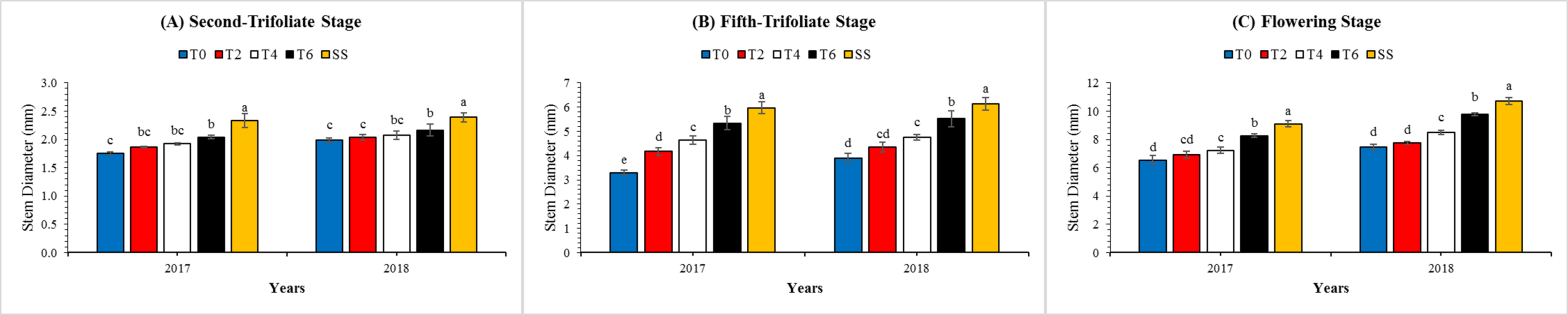
Changes in the stem diameter of soybean plants as affected by leaf excising treatments during 2017 to 2018 growing season. (A), (B) and (C) refer to second-trifoliate stage (V_2_), fifth-trifoliate stage (V_5_), and flowering-stage (R_1_), respectively. The T0, T2, T4, and T6 represent the no removal, removal of two, four, or six leaves, respectively, from maize canopy under relay-intercropping system. The SS refer to sole cropping system of soybean. Means are averaged over three replicates. Bars show ± standard errors, (*n* = 3). Within a bar, different lowercase letters show a significant difference (*P* ≤ 0.05) between treatments.

These observed measurements supported the quantitative determination of morphological parameter changes between the MS_R_ and SS. The plant height (Table 4) and lodging rate (Table 4) of soybean plants under MS_R_ were significantly (*P* < 0.05) higher than SS at all stages. Meanwhile, the opposite results were found for stem breaking strength and stem diameter. However, leaf removal treatments in MS_R_ considerably improved the growth of soybean plants by decreasing the plant height and lodging rate in both years. Overall, the plant height and lodging rate at R_1_ stage were decreased by 5%, 13%, and 23%, and 18%, 37%, and 59% in T2, T4, and T6 than T0 treatment under MS_R_, whereas, the stem breaking strength (Fig. 4) and stem diameter (Fig. 5) of soybean plants under T2, T4, and T6 increased by 15%, 28%, and 47%, and 4%, 11%, and 22%, respectively as compared to T0 treatment. The changes in plant height, lodging rate, stem diameter, and stem breaking strength at V_2_ and V_5_ under all treatments followed a similar pattern with those at R_1_ stage. Furthermore, at R_1_, the interactive effect of leaf excising treatments and year for plant height (Table 4), lodging rate (Table 4) and stem breaking strength (Table 3) was found non-significant, while it was found significant for stem diameter (Table 3).

### Leaf area index and total biomass accumulation

**Figure 6:**
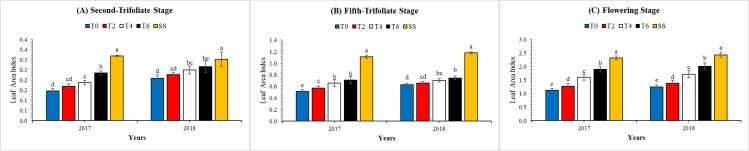
Variations in the leaf area index of the soybean plants as affected by leaf excising treatments during 2017 to 2018 growing season. (A), (B) and (C) refer to second-trifoliate stage (V_2_), fifth-trifoliate stage (V_5_), and flowering-stage (R_1_), respectively. The T0, T2, T4, and T6 represent the no removal, removal of two, four, or six leaves, respectively, from maize canopy under relay-intercropping system. The SS refer to sole cropping system of soybean. Means are averaged over three replicates. Bars show ± standard errors, (*n* = 3). Within a bar, different lowercase letters show a significant difference (*P* ≤ 0.05) between treatments.

The LAI showed significant (*P* < 0.05) variations from V_2_ to R_1_ of soybean, under different leaf removal treatments. In both study years, the LAI was enlarged rapidly from V_2_ to R_1_ of soybean and reached to highest value at R_1_ of soybean. Across the treatments, at R_1_ stage, the mean highest soybean LAI under SS was 101%, 79%, 43%, and 21% higher than those of under T0, T2, T4, and T6, respectively (Fig. 6). Interactive effect of leaf excising treatments and year for LAI was noted significant at V_2_ and non-significant at V_5_ and R_1_ (Table 3).

Furthermore, different leaf excising treatments significantly affected the total biomass (g plant^−1^) production of soybean at different sampling stages in both years (Table 4). In this experiment, SS always produced higher biomass as compared to different leaf removal treatments under MS_R_. However, the treatments T4 and T6 resulted in average higher biomass of soybean (16.5 and 20.1 g plant^−1^, respectively) at R_1_ stage, than other treatments in relay-intercropping. The total biomass accumulation of soybean plants exhibited the trend SS > T6 > T4 > T2 > T0 (Table 4). For example, at R_1_ stage, for treatment T6, it increased the biomass of soybean by 76% during the planting seasons as compared to T0. Interactive effect of leaf excising treatments and year for total biomass was found significant and non-significant at V_2_, and V_5_ and R_1_, respectively (Table 3). In this experiment, we also measured the distribution of biomass among different plant parts of soybean and we observed that the leaf removal treatments changed the pattern of biomass distribution between root, stem, and leaves of soybean plants (Table 6). At V_2_ and V_5_, the highest distribution of biomass was measured in stem followed by leaves and root under T0 and T2 treatment, whereas, the increasing light intensity treatments T4 and T6 translocated the maximum amount of biomass to leaves (photosynthetic parts) than stem and root of soybean plants. Moreover, the biomass distribution in photosynthetic parts was increased substantially at R_1_, and the mean maximum biomass distribution in leaves (13.0 g plant^−1^) was found in SS. However, the leaf removal treatments accelerated the biomass allocation to leaves, and the highest biomass allocation to leaves 7.0, 8.9, and 10.7 g plant^−1^ was observed in T2, T4, and T6, respectively (Table 6). On average, at R_1_ stage, root, leaves and stem biomass increased by 45%, 36% and 59% under T4 and 75%, 98% and 63% under T6 in comparison with T0, respectively. Moreover, the interactive effect of leaf excising treatments and year for biomass distribution to roots, stem, and leaves was found non-significant at R_1_ stage (Table 6).

**Table 6:** Effects of leaf excising treatments on total biomass accumulation and distribution in root, stem, and leaves of soybean plants at second-trifoliate stage (V_2_), fifth-trifoliate stage (V_5_), and flowering-stage (R_1_) under sole cropping and relay intercropping system from 2017 to 2018.

Years	Treatments	Biomass Distribution (g plant^−1^)								
		V_2_			V_5_			R_1_		
		Roots	Stem	Leaves	Roots	Stem	Leaves	Roots	Stem	Leaves
2017	T0	0.10^d^	0.52^d^	0.49^c^	0.31^e^	2.22^c^	1.83^c^	0.70^e^	3.88^d^	5.70^d^
	T2	0.12^c^	0.59^c^	0.56^b^	0.34^d^	2.39^bc^	2.05^c^	0.85^d^	4.97^cd^	6.65^d^
	T4	0.12^bc^	0.63^b^	0.57^b^	0.38^c^	2.42^bc^	2.64^b^	1.07^c^	6.30^bc^	8.36^c^
	T6	0.13^b^	0.67^ab^	0.58^b^	0.43^b^	2.57^b^	3.06^b^	1.27^b^	7.47^b^	9.95^b^
	SS	0.15^a^	0.69^a^	0.80^a^	0.51^a^	2.96^a^	3.79^a^	1.63^a^	9.96^a^	12.42^a^
	LSD	0.00	0.04	0.05	0.03	0.33	0.49	0.13	1.55	1.56
2018	T0	0.12^e^	0.66^b^	0.56^d^	0.34^e^	2.43^b^	2.03^c^	0.85^d^	4.28^d^	7.37^d^
	T2	0.13^d^	0.74^a^	0.62^d^	0.37^d^	2.67^ab^	2.17^c^	0.93^d^	5.30^cd^	7.39^d^
	T4	0.15^c^	0.76^a^	0.74^c^	0.43^c^	2.54^ab^	3.14^b^	1.18^c^	6.63^c^	9.45^c^
	T6	0.16^b^	0.77^a^	0.88^b^	0.48^b^	2.87^ab^	3.43^b^	1.44^b^	8.68^b^	11.37^b^
	SS	0.18^a^	0.81^a^	0.99^a^	0.55^a^	3.17^a^	4.15^a^	1.77^a^	10.40^a^	13.53^a^
	LSD	0.00	0.08	0.06	0.03	0.69	0.35	0.16	1.56	1.86
ANOVA										
Year (Y)		*	*	*	*	NS	NS	*	NS	NS
Treatment (T)		*	*	*	*	*	*	*	*	*
Interaction (Y × T)		*	NS	*	NS	NS	NS	NS	NS	NS

**Notes:**

The T0, T2, T4, and T6 represent the no removal, removal of two, four, or six leaves, respectively, from maize canopy under relay-intercropping system. The SS refer to sole cropping system of soybean. Means are averaged over three replicates. Means that do not share the same letters in the column differ significantly at *P* ≤ 0.05.

NS, non-significant; *, significant.

### Seed yield

**Figure 7:**
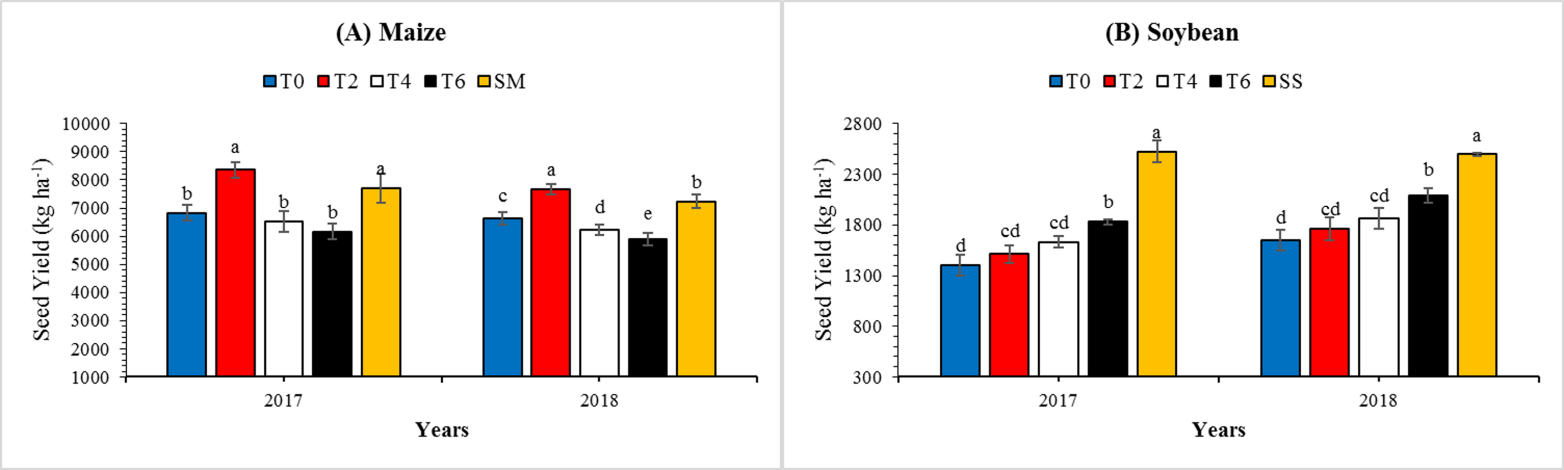
Variations in the seed yield of maize (A) and soybean (B) as affected by leaf excising treatments during 2017 to 2018 growing season. The T0, T2, T4, and T6 represent the no removal, removal of two, four, or six leaves, respectively, from maize canopy under relay-intercropping system. The SS refer to sole cropping system of soybean. Means are averaged over three replicates. Bars show ± standard errors, (*n* = 3). Within a bar, different lowercase letters show a significant difference (*P* ≤ 0.05) between treatments.

Different leaf excising treatments led to significant (*P* < 0.05) differences in seed yields of maize and soybean under MS_R_ (Fig. 7). By averaging the 2 years data, we observed that the seed yield of soybean in T0, T2, T4, and T6 under maize soybean relay intercropping system was 61%, 65%, 70%, and 78% of that in SS, respectively. Among all the leaf excising treatments in MS_R_, T6 had the highest mean soybean seed yield (1,961.4 kg ha^−1^) while T0 had the lowest seed yield (1,528.3 kg ha^−1^). Furthermore, seed yield of maize under treatment T0, T2, T4, and T6 in MS_R_ was 90%, 107%, 85%, and 81% of that in sole maize seed yield (Fig. 7). Overall, as compared to T0, seed yields of soybean increased by 15% and 28%, and maize decreased by 6% and 12% under treatment T4 and T6, respectively in both study years. Furthermore, the interactive effect of leaf excising treatments and year for seed yield of maize and soybean was found non-significant and significant, respectively (Table 3).

### Correlation analysis

**Figure 8:**
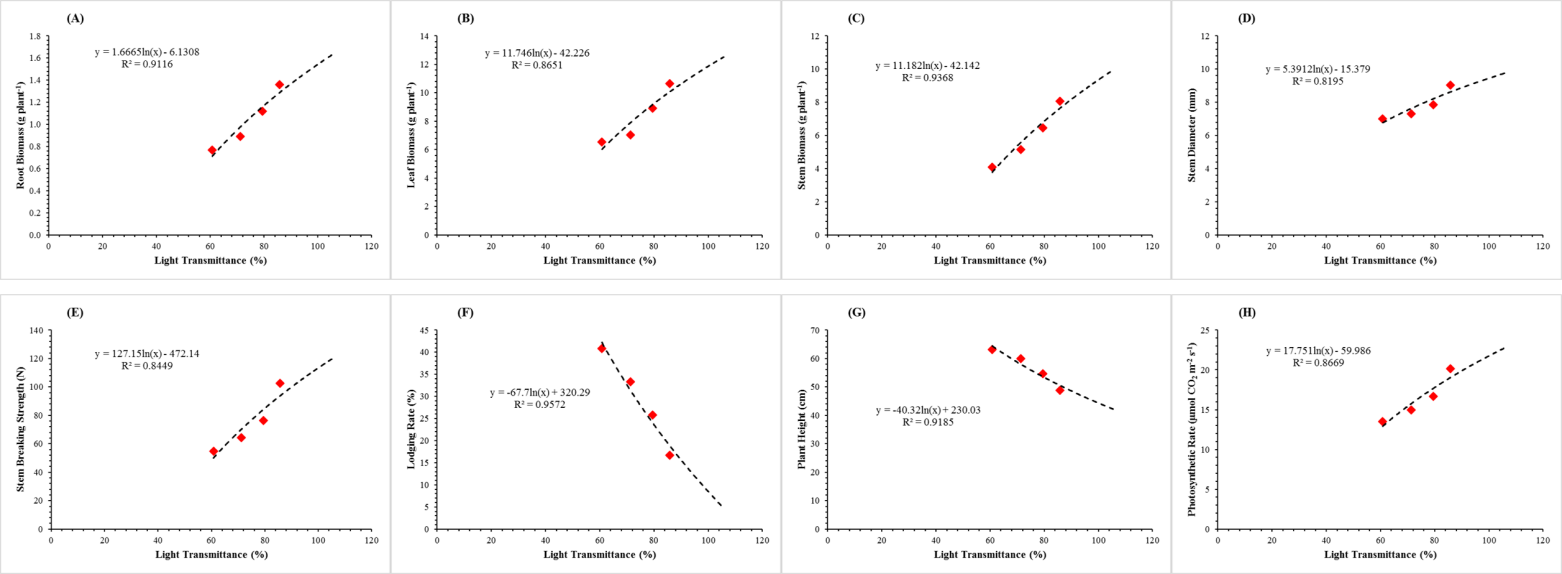
Relationship of the light transmittance with the root biomass (A), leaf biomass (B), stem biomass (C), stem diameter (D), stem breaking strength (E), lodging rate (F), plant height (G), and photosynthetic rate (H) at R_1_ stage of soybean.

To recognize the indices wherein soybean growth was sensitive to available light transmittance (PAR_T_), the relationship between the increasing light transmittance and soybean growth characteristics were investigated (Fig. 8). Among the morphological parameters of soybean, the stem diameter, lodging resistance and stem breaking strength of soybean increased with the increased light transmittance, while the plant height of soybean plants was decreased. We found that root biomass (*R*^2^ = 0.91; Fig. 8A), leaf biomass (*R*^2^ = 0.86; Fig. 8B) and stem biomass (*R*^2^ = 0.94; Fig. 8C), stem diameter (*R*^2^ = 0.82; Fig. 8D) and stem breaking strength (*R*^2^ = 0.85; Fig. 8E) at R_1_ were strongly and positively (*P* < 0.05) related with the increasing light transmittance at the top of soybean canopy. Whereas, a strong negative relationship was observed between the increasing light transmittance and decreasing lodging rate (*R*^2^ = 0.96; Fig. 8F) and plant height (*R*^2^ = 0.92; Fig. 8G). In addition, the photosynthetic rate (*R*^2^ = 0.87; Fig. 8H) at R_1_ also had a strong and positive relationship with increasing light transmittance at the top of soybean canopy. The correlation coefficient between all the measured parameters and increasing light transmittance for the mean datasets of 2017 and 2018 were all higher than 0.82 (*P* < 0.05).

## Discussion

### Light quality and intensity

Solar radiations are the most important environmental factor for agricultural crops (Aphalo, Ballare & Scopel, 1999). In fields, crop plants often experience shading conditions especially under intercropping conditions (Li et al., 2014). Shading condition decreases the light intensity and impairs the light quality of solar radiations (Park & Runkle, 2017). Similarly, in our study, maize shading significantly decreased the red to far-red light (R:FR) ratio for soybean under MS_R_; however, leaf excising treatments improved the light quality by increasing the R:FR ratios (Fig. 3). Specifically, the R:FR ratio in MS_R_ under T6 were from 1.11 to 1.35, which was considerably higher than T0 and nearly equal to that of SS. This increase in R:FR ratio was might be due to the reduced shade of maize at soybean canopy because previously scientists have confirmed that the R:FR ratio reduces from 1.2 in natural light to 0.05 under dense canopies, with a considerable decrease occurring before the canopy closure (Smith, 2000).

Moreover, the light intensity (PAR_T_) at the top of soybean in SS and MS_R_ changed considerably because the incident solar radiations absorbed and reflected by maize upper leaves could decrease the amount of available PAR for soybean plants under MS_R_ (Table 2). While in our study, leaf excising treatments in MS_R_ increased the PAR_T_ at the top soybean from 36% in T0 to 64% in T6 because the leaf excising from the top of maize plants decrease the rate of PAR reflected and absorbed by maize leaves, and increase the percentage of incident PAR at the top of soybean plants in MS_R_. The increase in PAR_T_ at the top of soybean canopy in MS_R_ can improve the soybean seedling growth during the co-growth period. Similarly, the positive impact of leaf excising treatments was observed on the PAR_T_ at the middle leaves of maize (Liu et al., 2017a; Xue et al., 2017). Therefore, by altering the maize canopy under maize soybean intercropping conditions we can increase the light intensity and improve the light quality at soybean canopy.

### SPAD values and photosynthetic parameters

Sunlight influences the plant growth processes; by the process of photosynthesis, plants utilize sun light to change water and CO_2_ into sugar, chlorophyll content play a vital role in converting the sunlight energy to chemical energy (Liang, 2000; Yuncong, Shaohui & Yun, 2007). Under shading conditions, the investigation of chlorophyll content helps as an index for light absorption (Fan et al., 2018). Previously, it has been reported that chlorophyll content is greatly affected by light environment (Feng et al., 2018; Khalid et al., 2019; Odeleye, Togun & Tayo, 2001; Sun & Weng, 2010). In our experiment, leaf excising treatments significantly improved the SPAD values of soybean leaves in MS_R_. This increase might be due to the improved light environment (Feng et al., 2018), reduced shade (Fan et al., 2018) and optimum growing conditions at soybean canopy that probably increased the SPAD values of soybean leaves in MS_R_ (Table 4).

The soybean leaves are responsive to shading conditions, and reduce light condition decreases the photosynthetic rate of soybean in MS_R_ (Jiang et al., 2011; Yang et al., 2017). In the present experiment, increased light intensity at soybean canopy from T2 to T6 led to increasing the photosynthetic and transpiration rate of soybean plants, but the stomatal conductance and intercellular CO_2_ level decreased at V_2_, V_5_, and R_1_ stages of soybean plants (Table 5), suggesting that reduce shade and increase PAR transmittance at soybean canopy can improve the photosynthetic characteristics of soybean plants in MS_R_. Our findings are similar with previous reports in which scientists have confirmed that crops change their photosynthetic characteristics to adapt to changing light conditions (Dai et al., 2009; Huang et al., 2011) and the optimum excising of leaves from canopy increased the photosynthetic rate of crop plants by increasing the light transmittance at middle strata leaves (Liu et al., 2017a). Consequently, this suggested that the reduced photosynthetic rate of soybean plants under intercropping systems are caused by severe maize shading (Yang et al., 2017). Therefore, this indicated that treatments T4 and T6 exhibited the improved photosynthetic characteristics than treatment T0 in maize soybean relay-intercropping system.

### Morphological characteristics

Leaf excising treatments from the top of maize plant in MS_R_ was set up to improve the light intensity (PAR) and quality (R:FR ratio) of soybean plants, to benefit the seedling growth and stem breaking strength and therefore to reduce the lodging rate of soybean plants. The changes in light quality and intensity can initiate morphological responses in crops (Kurepin et al., 2007). Soybean plants growing under the intercropping system are normally receiving altered light quality and quantity (Yang et al., 2014) and lower light intensity and R:FR ratio usually increased the plant height (Smith, 2000) and reduced the lodging resistance (Liu et al., 2016). In our experiment, similar results were observed that the increased light intensity with improved R:FR ratio at the top of soybean canopy led to decreased the plant height (Table 4) and lodging rate (Table 4) of soybean plants in MS_R_. Therefore, under enhanced light conditions, growth and development of soybean plants were improved which eventually decreased the soybean lodging in MS_R_ (Liu et al., 2016). Moreover, a positive correlation was measured between the increasing light transmittance and morphological characteristics of soybean plants except for lodging rate (Fig. 8F) and plant height (Fig. 4G). Our findings are similar to the notion that soybean plant height and lodging rate increased with the increase in shade intensity under relay-intercropping conditions (Morelli & Ruberti, 2002; Nagasuga & Kubota, 2008).

In addition, the stem breaking strength and stem diameter of soybean plants is of major concern because these parameters were decreased significantly by maize shade in MS_R_ (Liu et al., 2016; Wu, Gong & Yang, 2017). These plant characters directly affect soybean lodging and growth during the co-growth period (Yan et al., 2010). Interestingly, the results of our experiment revealed that leaf excising treatments considerably decreased the shade on soybean plants, increased the stem breaking strength (Fig. 4) and stem diameter (Fig. 5) of soybean plants in MS_R_. Results of this experiment are similar with previous findings which concluded that shade decreases the stem breaking strength and stem diameter of soybean seedlings in the relay-intercropping system of maize and soybean (Liu et al., 2016). Hence, increased stem breaking strength and stem diameter at seedling stage of soybean were more conducive to decrease the soybean lodging in MS_R_. Overall, these results showed that the soybean response to different leaf excising treatments under T0, T2, T4, and T6 is the combined effect of light intensity and quality, and by managing the maize canopy shade to an optimum level we can improve morphological parameters of soybean plants under MS_R_.

### Leaf area index and biomass accumulation

Soybean is a C_3_ crop that possesses lower photosynthetic rate than C_4_ plants (Jiang et al., 2011). Increase in LAI can enhance radiation use efficiency (Raza et al., 2019b) and growth by enhancing the photosynthetic activity (Liu et al., 2017b). At all growth stages (V_2_, V_5_, and R_1_), the LAI of soybean plants obtained maximum value under treatment T6 followed by T4, T2, and T0 (Fig. 6). Higher LAI resulted from increased PAR transmittance shows the optimum leaf development and expansion, which facilitated the soybean leaves in better capturing and utilization of solar radiation (Raza et al., 2019b). This increment in LAI was may be due to the increased light intensity at soybean canopy and leaf excising from the top of maize plant significantly increased the PAR transmittance at soybean canopy.

This study also provides data of biomass accumulation in soybean plants under increasing light intensity conditions (Table 4). Increased photosynthetic-activity is one of the main factors for biomass production (Raza et al., 2018b). It had been demonstrated that reduced light transmittance and impaired light quality for soybean in MS_R_ limited the growth potential and produced the less biomass (Wu, Gong & Yang, 2017; Yang et al., 2014). Our findings proposed that leaf excising from the top of maize plants during the co-growth phase of maize and soybean in MS_R_, soybean plants sufficiently captured and utilized light for their physiological and biochemical processes and maintained the higher biomass production which in turn improved the morphological characteristics especially reduced the lodging rate. Our findings are similar to previously published results (Yang et al., 2018b). We further evaluated the biomass distribution in root, stem, and leaves of soybean in response to leaf excising treatments (Table 6). The biomass distribution changed considerably at V_2_, V_5_, and R_1_ in soybean plants. At all growth stages (V_2_, V_5_, and R_1_), in treatment T0, highest distribution of biomass was noted in stem followed by leaves and roots, while under T2, T4, and T6 treatments higher translocation of biomass was measured in leaves followed by stem and roots (Table 6). The improved light environment at the top of soybean canopy increased the uniform distribution of biomass among roots, stem, and leaves in MS_R_ (Ahmed et al., 2018; Feng et al., 2019; Raza et al., 2019a). Whereas, under shading conditions, the major part of soybean biomass was used in stem elongation to capture more sun light which ultimately enhanced the rate of soybean lodging in MS_R_ (Liu et al., 2016; Wu, Gong & Yang, 2017; Yang et al., 2014). The improve light intensity significantly increased the biomass of roots and under intercropping conditions root interactions can play an important role when the availability of water and nutrient are more limited than light (Mushagalusa, Ledent & Draye, 2008). In this experiment, we also measured the impacts of leaf excising treatments on the seed yields of maize and soybean in MS_R_. Results exhibited that the maximum (2,509.9 kg ha^−1^) seed yield of soybean was noted in SS, while among leaf excising treatments in MS_R_, the highest soybean seed yield was obtained in T6 (1,961.5 kg ha^−1^) followed by T4 (1,750.4 kg ha^−1^), T2 (1,636.9 kg ha^−1^) and T0 (1,528.2 kg ha^−1^) in study both years, with an improvement of 30% in 2017 and 27% in 2018 under T6 as compared to T0 (Fig. 7B). Furthermore, the seed yield of maize under treatments T0, T2, T4, and T6 in MS_R_ was 90%, 107%, 85%, and 81% of that in SM seed yield (Fig. 6A), suggesting that excising leaves from the top of maize plants after silking stage can increase the seed yield of soybean plants under MS_R_ by maintaining the maize yield. In addition, leaf excising treatments significantly improved the light environment which in turn considerably increased the photosynthetic rate of soybean plants during the co growth period (Table 5). Although excising of leaves from the top maize plants reduced the seed yield of maize plants by 6% in T4 and 12% in T6 than T0 under MS_R_, but it increased the seed yield of soybean by 15% in T4 and 28% in T6 in both years. Therefore, it is new sustainable agronomic-approach to reduce the adverse impacts of maize shading on the growth and development of soybean in MS_R_. Overall, the excising of leaves from the top of maize plants under MS_R_ significantly improves the morphological and photosynthetic characteristics of soybean plants, accelerated the biomass production during the cogrowth phase in MS_R_ and compensated the yield loss of maize by considerably increasing the soybean yield (Fig. 7B). Taken together, our results of the present experiment confirmed that leaf excising from maize top after silking stage considerably enhanced the available light for soybean plants which increased the biomass accumulation and lodging resistance of soybean plants under maize soybean relay-intercropping system. Therefore, PAR transmittance at soybean canopy is an important factor to obtain healthy soybean plants in MS_R_.

## Conclusion

The study reported in this paper was designed to investigate the effects of leaf excising treatments on the light environment of soybean because in MS_R_ soybean experience severe shading conditions especially from the germination stage to flowering stage. Based on our results, we demonstrated that different leaf excising treatments from the top of maize plants had positive effects on the light environment of soybean plants under relay intercropping system. The morphological and photosynthetic characteristics were improved for all leaf excising treatments. Moreover, we noticed that the central strategy of soybean plants to cope shading conditions in MS_R_ was the uniform distribution of biomass among roots, stem, and leaves which can be maintained by leaf excising treatments under relay intercropping system or this could be achieved by developing the maize varieties with fever leaves above than ear. In addition, for the sustainability of maize soybean relay intercropping system, environment-friendly agronomic approaches are needed to improve the seedling growth of soybean plants in MS_R_, and our results of this study provide the new insights into the impacts of leaf excising treatments in MS_R_.

## Supplemental Information

10.7717/peerj.7262/supp-1 Supplemental Information 1 The raw data of all the measured parameters in this study from 2017 to 2018.

## Additional Information and Declarations

### Field Study Permissions

The following information was supplied relating to field study approvals (i.e., approving body and any reference numbers): All experiments were performed according to institutional guidelines of Sichuan Agricultural University, China.

### Data Availability

The following information was supplied regarding data availability: Raw data is available as a Supplemental File.
